# Optimization and assessment of a sequential extraction procedure for calcium carbonate rocks

**DOI:** 10.1007/s10661-021-09300-x

**Published:** 2021-08-16

**Authors:** Janin Scheplitz, Sarah Koopmann, Henning Fröllje, Thomas Pichler

**Affiliations:** Fachbereich Geowissenschaften, Universität Bremen, Klagenfurter Str. 2-4, 28359 Bremen, Germany

**Keywords:** Sequential extraction analysis, Carbonate, Contaminant, Karst aquifer, Molybdenum, Arsenic

## Abstract

Sequential extraction analyses are widely used for the determination of element speciation in sediments and soils. Typical sequential extraction protocols were developed to extract from low-carbonate samples and therefore are not necessarily suitable for high-carbonate samples. In this study, we tested increased reagent to sample ratios to adjust an existing sequential extraction procedure to analyze high-CaCO_3_ samples with concentrations ranging from 70 to above 90 %. Complete dissolution of the CaCO_3_ phase, and a higher extraction efficiency of manganese associated with the carbonate phase, was achieved when using four times the original reagent to sample ratio in the 2nd extraction step. This increase of reagent did not compromise the extraction of subsequent phases as shown by unaffected Fe concentrations in a low-carbonate sample. Hence, an essential outcome was that increasing the solution to sample ratio did not lead to the dissolution of other sedimentary phases, such as hydrous and crystalline iron oxides or sulfides. Thus, compared to other extraction protocols that use a lower reagent to sample ratio in the carbonate dissolution step, the new protocol allowed the complete extraction of oxide and sulfide phases in the following extraction steps. Furthermore, the study demonstrated the benefit of replacing Na-acetate with NH_4_-acetate to extract exchangeable ions and carbonates. We observed increased intensities for several analytes, i.e., trace metals such as Mo and As, due to less suppression of the analyte signal by NH_4_-acetate than by Na-acetate during analysis by inductively coupled plasma optical emission spectrometry (ICP-OES).

**Supplementary information:**

The online version contains supplementary material available at 10.1007/s10661-021-09300-x.

## Introduction

Carbonate aquifers supply approximately 20 to 25 % of the world’s population with water (Ford & Williams, 2007; Goldscheider et al., 2020). While they are generally great for water quantity, they can be problematic with respect to water quality. Already at minute degrees of karstification, rapid flow through larger voids can negate the purification properties that generally make groundwater a safe source of drinking water (e.g., Katz, 2004; McMahon et al., 2008). Hence, understanding water-rock interaction and the associated release of contaminants in carbonate aquifers is essential to evaluate and forecast possible contamination scenarios. The importance of investigations into carbonate aquifers concerning the release of pollutants, such as molybdenum (Mo) or arsenic (As), has been demonstrated (Jones & Pichler, 2007; Katz et al., 2009; Lazareva et al., 2015; Pichler & Mozaffari, 2015). Anthropogenic influences can play a critical role in this context, as shown in central Florida, where drilling activities led to the injection of oxygen-rich water into a deep, anoxic carbonate aquifer (Wallis & Pichler, 2018). Consequently, through the induced oxidation of organic matter, large amounts of up to 4740 μg/L Mo and 371 μg/L As were released; both exceeding the World Health Organization mandated health-based threshold values for drinking water of 70 μg/L for Mo and 10 μg/L for As (Pichler et al., 2017; WHO, 2011).

An integral approach to understanding the mobility of trace metals in the shallow subsurface is sequential extraction analysis, which allows conclusions about the chemical bonding of elements in the aquifer matrix (e.g., Filgueiras et al., 2002). Combined with complementary methods such as batch experiments, mobilization processes of contaminants to ambient water can be estimated (e.g., Koopmann et al., 2019). The basic principle of the sequential extraction procedure of sediments or soils is the differentiation of the analytes’ chemical bonding by step-wise extraction using different reagents. The choice of reagent depends on the particular research questions and the type of material under investigation. Based on the classic sequential extraction method of Tessier et al. (1979), some well-established extraction protocols were developed (e.g., Gleyzes et al., 2002; Quevauviller et al., 1994; Rauret et al., 1999; Sutherland & Tack, 2002; Ure et al., 1993a). There have been approaches to extract metals from high-carbonate samples (Gleyzes et al., 2001; Orsini & Bermond, 1993), but none was optimized for extraction from a dominantly calcium carbonate (CaCO_3_) matrix. While the focus was often the extraction of As from different soils and sediments (e.g., Gleyzes et al., 2001; Keon et al., 2001; Price & Pichler, 2005; Pichler & Veizer, 2004; Romero et al., 2003; Van Herreweghe et al., 2003; Wenzel et al., 2001), only a few were optimized for the extraction of Mo (Aydin et al., 2012; Liang & Zhu, 2016; Zemberyova et al., 2010).

Sequential extraction procedures were developed to extract from heterogeneous sediments and soils (e.g., Quevauviller et al., 1994; Tessier et al., 1979), generally consisting of carbonate, hydroxide, oxide, silicate, sulfate, and sulfide minerals. The difficulty of performing sequential extraction analyses of carbonate sediments is that they are relatively homogenous, consisting of mainly CaCO_3_ minerals, such as calcite and aragonite. Thus, although present at concentrations above 80 %, it is crucial that the carbonate phase is extracted in a discrete step and not as part of the following steps. Here, we present a modification of an established sequential extraction procedure originally developed by Hall et al. (1996) and Pichler et al. (2001) for the application to heterogeneous soils and sediments. The main objective of our study was to develop a sequential extraction procedure for carbonate-rich samples that guarantees the quantitative dissolution of calcium carbonate in a discrete carbonate-removal step. This is crucial for the extraction of potential contaminants (e.g., As, Mo) together with their associated host phases, since incomplete dissolution of carbonates has the potential to buffer the extraction of the target phases in the following extraction steps. Similarly, excess extraction solution may unintentionally attack oxide or sulfide phases. Hence, besides aiming for the complete dissolution of carbonates in a discrete step, an additional focus was to avoid compromising the subsequent extraction steps, particularly those of hydrous and crystalline iron oxides.

## Experimental

### Sample material

The optimization of the sequential extraction procedure was carried out using four CaCO_3_ sediment samples (C-90, C-68, C-78, C-74, Table 1) from two cores (DEP-1 and DEP-2) drilled in the municipality of Lithia in central Florida (Pichler & Mozaffari, 2015; Pichler et al., 2017). The samples cover the Hawthorn Group, a lithostratigraphic unit of the Intermediate Aquifer System, which was deposited in a shallow marine to non-marine fluvial depositional environment mainly during the Miocene. Detailed descriptions of the lithostratigraphy and hydrostratigraphy of the area can be found elsewhere (Katz et al., 2009; Miller, 1986; Pichler & Mozaffari, 2015; Pichler et al., 2011). The samples were primarily chosen based on their CaCO_3_ contents, which were determined with a calcium carbometer and ranged from 68 to 90 % (Table 1). In addition to the Floridian samples, a certified reference material GBW-07120 (China National Analysis Center) and a shale sample with approximately 14 % CaCO_3_ (PS-14) were analyzed as well (Table 1).

**Table 1:** Samples used for the optimization of the sequential extraction procedure

**Sample**	**Material**	**Origin/core**	**Lithology**	**Depth** **(m)**	**CaCO**_**3**_ **(%)**
C-90	Limestone	DEP-1	Miocene, Hawthorn Group	136	90
C-68	Limestone	DEP-1	Miocene, Hawthorn Group	152	68
C-78	Limestone	DEP-2	Miocene, Hawthorn Group	105	78
C-74	Limestone	DEP-2	Miocene, Hawthorn Group	112	74
PS-14^a^	Posidonia Shale	Rheden 6 Core 19	Lower Jurassic, Toarcian	1603 -1605	14
GBW-07120	Limestone	GBW-07120	Certified Reference Material	-	92

^a^S. Koopmann, unpublished data, samples provided by Wintershall Dea GmbH, Germany

All samples were crushed in an agate mortar and subsequently ground to a fine powder in a planetary micro mill (Fritsch Pulverisette 7).

### Reagents

All solutions were prepared using ultrapure water (18.2 MΩ, Milli-Q). Analytical-grade Na-acetate (NaCH_3_COO; AppliChem, Germany), reagent-grade NH_4_-acetate (NH_4_CH_3_COO; Fisher Chemical, USA), ReagentPlus®-grade hydroxylamine hydrochloride (NH_2_OH∙HCl; Sigma-Aldrich, Germany), and trace metal-grade acetic acid (CH_3_COOH; Fisher Chemical, USA) were used for the preparation of the extractants. Hydrochloric acid (HCl) and nitric acid (HNO_3_) were purified by sub-boiling from analytical-grade acids (Merck, Germany). Adjustment of pH was done using sub-boiled HNO_3_ and trace analysis supra-grade ammonia solution (NH_4_OH; Bernd Kraft, Germany).

### Aqua regia digestion

To determine the total extractable element content in the samples, we followed a procedure based on the modified BCR method (Rauret et al., 1999; Sutherland & Tack, 2002), where 10 mL aqua regia was added to 0.5 g of powdered sample and digested in a hot block. After being kept at room temperature overnight, the samples were heated to 120 °C for 2 h under reflux, diluted with ultrapure water to 50 mL after cooling and subsequently filtered using in-vial filters (Environmental Express Filter Mate, 6 µm pore size). Before the measurements, the samples were further diluted 1:1 with ultrapure water to achieve a 10 % acid matrix. Aqua regia digestion was done in triplicate for each sample. All samples were stored at room temperature until analysis.

### Sequential extraction procedure

#### Original method

The first set of sequential extraction procedures were performed using a slightly modified version of the extraction procedure published by Hall et al. (1996) and Pichler et al. (2001) (Table 2). That procedure was considered the base for the adjustments for the high and variable carbonate contents of the samples. Other than in the original procedure, the multi-acid digestion to dissolve silicates and residuals was not considered necessary for this study. The sequential extraction was done in triplicate for each sample.

**Table 2:** Slightly modified sequential extraction scheme based on Hall et al. (1996) and Pichler et al. (2001) (see section “Original method”) adjusted to 0.5 g of sediment material

**Step**	**Phase**	**Reagents**	**Procedure**
1	Adsorbed/exchangeable	10 mL 1.0 M NaCH_3_COO (pH 8.2)	2 h leach, 2 × 5 mL H_2_O rinse
2	Carbonates	10 mL 1.0 M NaCH_3_COO (pH 5.0)	2 h leach, 2 × 5 mL H_2_O rinse
3	Hydrous iron oxides	10 mL 0.25 M NH_2_OH∙HCl in 0.25 M HCl	2 h bath at 60 °C, 2 × 5 mL H_2_O rinse
4	Crystalline iron oxides	15 mL 1.0 M NH_2_OH∙HCl in 25 % CH_3_COOH	3 h bath at 90 °C, 2 × 5 mL H_2_O rinse
5	Sulfides/organic material	10 mL aqua regia (7.5 mL HCl, 2.5 mL HNO_3_)	~ 12 h bath (2 h at 120 °C)

The extraction sequence was carried out using 0.5 g of sediment, which was weighed directly into centrifuge tubes, which also served as the reaction vessels. After each extraction, the samples were centrifuged for 15 min, the extract decanted into a 50-mL polypropylene tube, filtered through a 0.45 µm pore size nylon filter, and acidified to 2 % HNO_3_. Before adding the next reagent, the residual sediment was washed and subsequently centrifuged twice with 5 mL ultrapure water. The fifth extraction step (sulfides/organic material) was done, as described in section “Aqua regia digestion.” All samples were done in triplicate to control potential uncertainties in the extraction efficiency.

In preparation for chemical analysis, the extracts from steps 1 and 2 were diluted 1:2 with 2 % HNO_3_, and those of extraction step 4 were diluted 1:1 with 2 % HNO_3_ to circumvent stability problems during the ICP-OES measurements that arise from high acetic acid/acetate matrix loads, as observed in preliminary measurements. All samples were stored at room temperature until analysis.

#### Adjustments to the sequential extraction procedure

The original sequential extraction procedure was modified to optimize the method for samples with high CaCO_3_ contents. We increased the reagent to sample ratio in the carbonate extraction step (step 2) to dissolve carbonates quantitatively while keeping the reagent to sample ratio constant in step 1 as well as in steps 3–5. As part of this adjustment, we lowered the sediment amount to 0.25 g to be able to increase the reagent to sample ratio with the available equipment (limitation to 20 mL solution). The adjustment of reagent to sample ratio was done using Na-acetate and NH_4_-acetate in extraction step 2 (see below). Due to the excellent reproducibility in our first set of analyses, we refrained from performing those extractions in triplicate. Simultaneously, we replaced Na-acetate by NH_4_-acetate in steps 1 and 2 and compared the extraction efficiencies to avoid high sodium loadings during ICP-OES measurements. For both extraction solutions, the pH was kept at the original value of pH = 8.2 for step 1 and pH = 5.0 for step 2 (Tables 2 and 3). The changes to the sequential extraction procedure are summarized in Table 3. All the samples were stored at room temperature until analysis.

**Table 3:** Modifications to the sequential extraction scheme shown in Table 2 and adjusted to 0.25 g of sediment material

**Step**	**Phase**	**Reagents**	**Procedure**
1	Adsorbed/exchangeable	5 mL 1.0 M NaCH_3_COO (pH 8.2) or 1.0 M NH_4_CH_3_COO (pH 8.2)	2 h leach, 2 × 5 mL H_2_O rinse
2	Carbonates	10 mL, 15 mL, or 20 mL 1.0 M NaCH_3_COO (pH 5.0) or 1.0 M NH_4_CH_3_COO (pH 5.0)	2 h leach, 2 × 5 mL H_2_O rinse
3	Hydrous iron oxides	5 mL 0.25 M NH_2_OH∙HCl in 0.25 M HCl	2 h bath at 60 °C, 2 × 5 mL H_2_O rinse
4	Crystalline iron oxides	7.5 mL 1.0 M NH_2_OH∙HCl in 25 % CH_3_COOH	3 h bath at 90 °C, 2 × 5 mL H_2_O rinse
5	Sulfides/organic material	5 mL aqua regia (3.75 mL HCl, 1.25 mL HNO_3_)	~ 12 h bath (2 h at 120 °C)

### ICP-OES analysis

Elemental concentrations of Ca (analytical wavelength: 317.933 nm), Mg (285.213 nm), Sr (407.771 nm), Fe (238.204 nm), Mn (257.610 nm), As (188.979 nm), and Mo (202.031 nm) were determined using the Optima 7300 DV inductively coupled plasma optical emission spectrometer (ICP-OES; Perkin Elmer). Instrumental setup and operational conditions are listed in Table 4. Calibration standards were prepared from 1000 mg/L single-element stock solutions (Inorganic Ventures, USA, and SPEX Certiprep, USA) and matrix-matched with the particular extraction step samples. Instrument blanks and procedural blanks were analyzed in addition to quality control standards. In-house tap-water and an artificial multi-element standard independently prepared from single-element stock solutions were used to check for accuracy, precision, and instrument drift during measurements. For analysis of the samples and standards, 5 mL of each solution was transferred to 10-mL PE vials and analyzed without further dilution using a standard sample introduction system (Table 4). All analyzed data were within the linear calibration range.

**Table 4:** Instrument setup and operational parameters of the ICP-OES analyses

**Nebulizer**	MiraMist PEEK
**Spray chamber**	Cyclonic glass
**View mode**	Radial and axial
**RF power (W)**	1500
**Plasma gas flow (L/min)**	15.0
**Torch position**	−2
**Auxiliary gas flow (L/min)**	1.0
**Nebulizer gas flow (L/min)**	0.8
**Pump rate (mL/min)**	1.5
**Rinse time (s)**	30
**Delay time (s)**	60
**Replicates per sample**	3

Instrument and procedural blanks were negligible for the analyzed elements in all extracted phases, and no instrument drift was observed during a daily run. Reproducibility of the analysis was checked by measurement of three replicates per sample having a deviation of generally < 2 %. Quality control standards agreed with the nominal values within an error of < 10 %. To assure the quality of the presented data, we do not report data for those measurements where the reproducibility (as relative standard deviation of signal intensities) of the ICP-OES replicate analysis (*n* = 3; Table 4) had an error of > 10 % (indicated as not determined (n.d.) in SI tables A2 to A5). This is typically the case for elements analyzed at very low signal intensities close to the blank intensity, i.e., elements with very low concentrations in the measurement solutions.

## Results

### Aqua regia digestions

The triplicate analyses were in good agreement, with an RSD of generally less than 5 % for those elements with concentrations above the detection limit (Table A.1, Supplementary Information (SI)). The carbonate content calculated from the Ca concentration, assuming that all Ca was from CaCO_3_, agreed with the concentration determined with the carbometer (Tables 1 and A.1, SI). Furthermore, the carbonate content of the reference material GBW-07120 calculated from the Ca analysis (91 %) agreed with the carbonate content determined with the carbometer (91 %) and carbonate content calculated from published CaO (51.1 %) and CO_2_ (39.8 %) values (China National Analysis Center). Overall, the results demonstrated that most Ca in the samples was from CaCO_3_ and thus allowed the use of the Ca concentration as a proxy.

### Quality control

Calcium contents in those samples for which triplicates existed (original method) were generally in excellent agreement, demonstrating the reproducibility of CaCO_3_ extraction (Table A.2, SI). Furthermore, calcium contents in the aqua regia digest compared to the sum of the five extractions steps were generally below 10 % (Tables A.2 and A.3, S1). Those results confirmed the suitability to extract the entire CaCO_3_ from the sediments in step 2.

### Assessment of reagent volumes

**Fig. 1:**
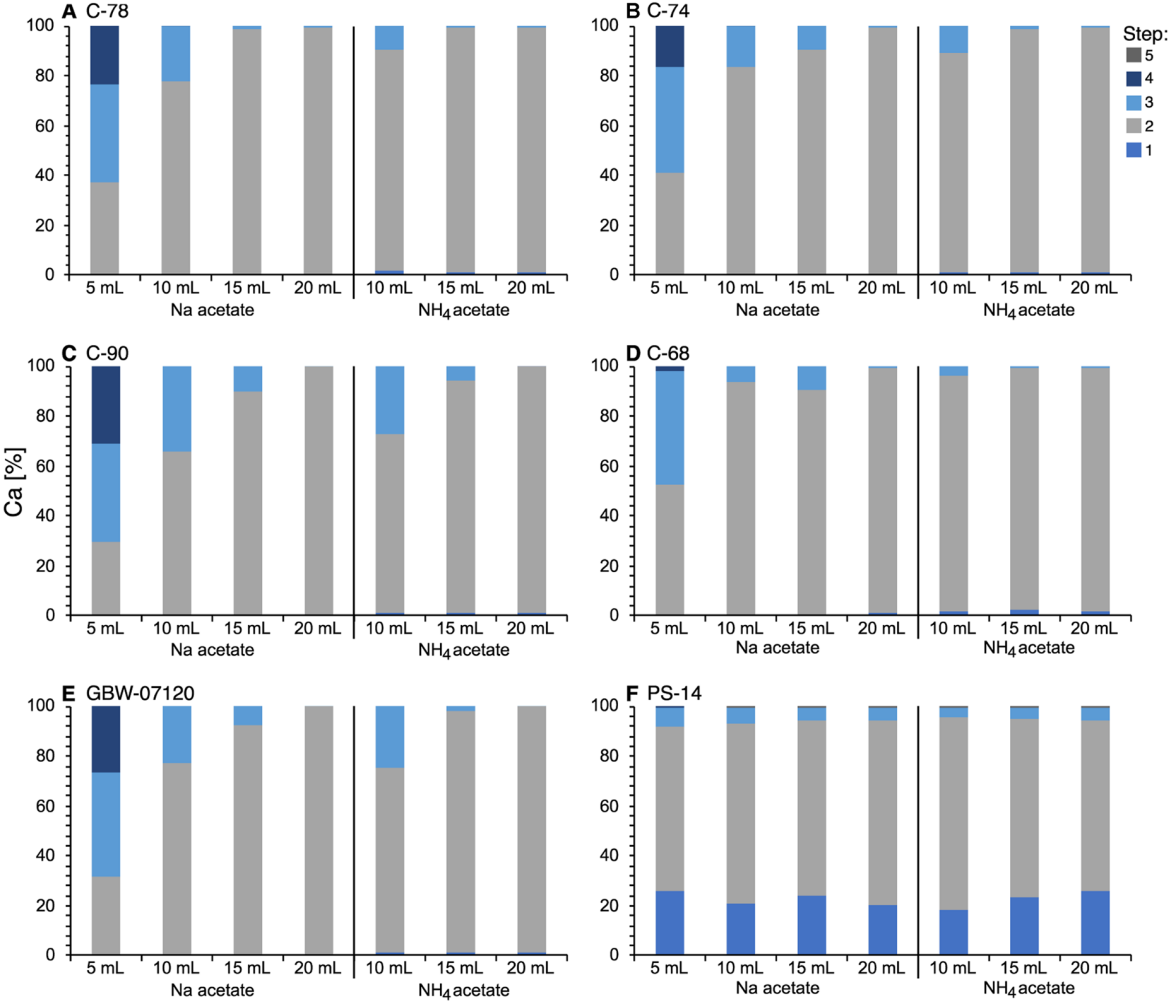
Ca (%) in extraction steps 1 to 5 for the original (5 mL NaOAc) and modified sequential extraction procedures (10, 15, and 20 mL, both NaOAc and NH_4_OAc)

To optimize the sequential extraction procedure for high CaCO_3_ samples, it was necessary to extract the CaCO_3_ entirely in extraction step 2. Results obtained with the original extraction procedure showed substantial amounts of Ca present in fractions 2, 3, and 4 (Fig. 1A–F, 5 mL). A closer look revealed that the sample with the lowest amount of Ca (C-68, Fig. 1D, 5 mL) had only little Ca left in fraction 4, whereas the samples higher in CaCO_3_ still had larger amounts of Ca in step 4.

Increasing the amount of reagent in extraction step 2 increased the amount of dissolved Ca in this step (Fig. 1A–F). A reagent volume of 20 mL caused a more or less complete extraction of Ca in step 2 and nearly no more Ca in steps 3 and 4. That observation was identical for both reagents, Na-acetate and NH_4_-acetate.

Calcium contents in the extractions made with Na- and NH_4_-acetate in extraction steps 1 and 2 were comparable for the same reagent volume (Fig. 1A–F; Table A.3, SI), although the amount of Ca in step 1 was slightly higher when using NH_4_-acetate (Table A.3, SI). A similar observation was made for Mg. However, during ICP-OES analyses, intensities were by up to 50 % higher when using NH_4_-acetate instead of Na-acetate in steps 1 and 2 (see “Discussion”).

**Fig. 2:**
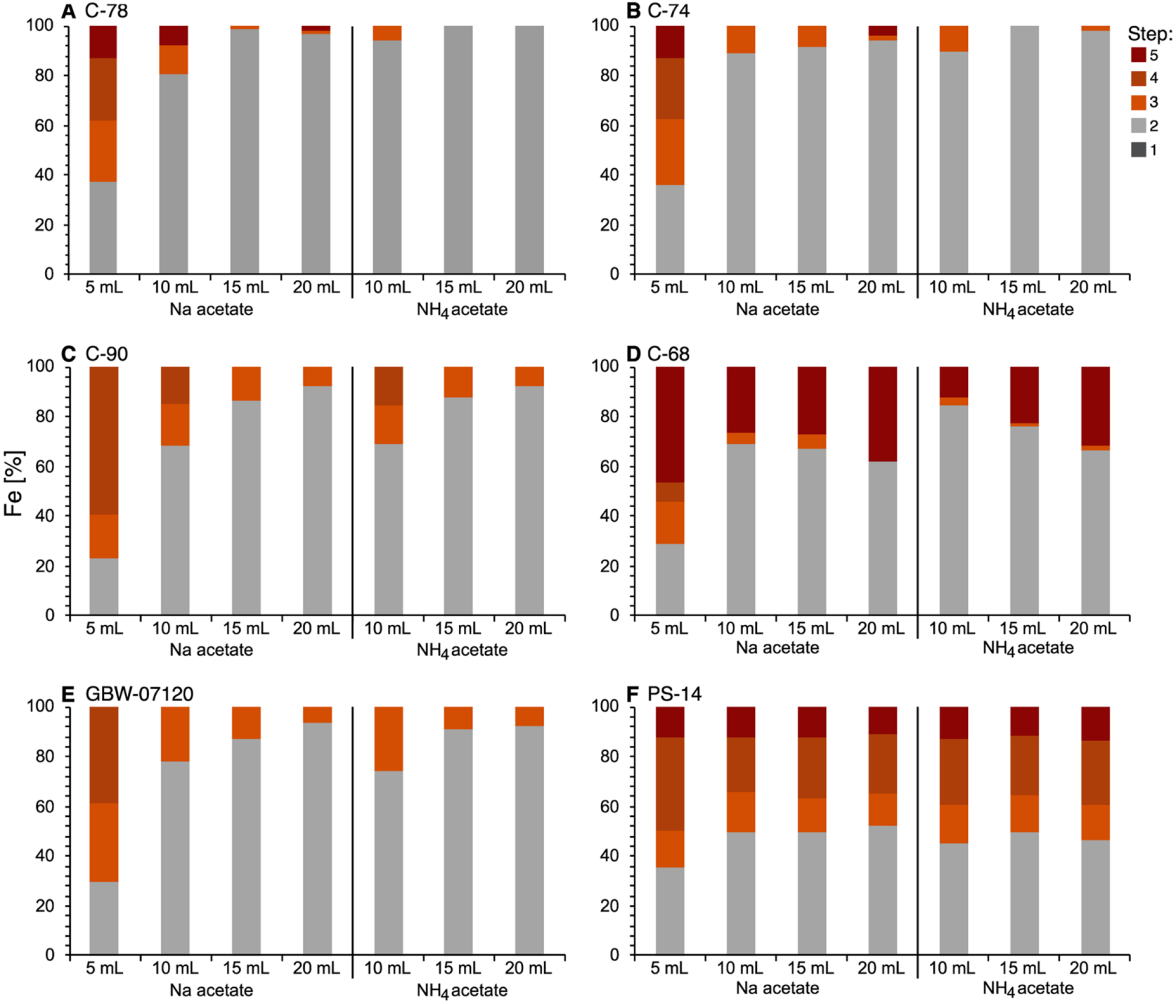
Mn (%) in extraction steps 1 to 5 for the original (5 mL NaOAc) and modified sequential extraction procedures (10, 15, and 20 mL, both NaOAc and NH_4_OAc)

Substantial amounts of Mn were found in steps 3 to 5 when the original extraction procedure was used (Fig. 2A–F). Similar to Ca, Mn was dissolved in extraction step 2 when increasing Na-acetate and NH_4_-acetate. In contrast to Ca, some samples (C-90, GBW) had some Mn left in step 3 in the adjusted protocol, while others showed complete removal of Mn in step 2 after increasing the reagent volume to 15 or 20 mL (C-78, C-74). For the Posidonia shale, we observed a slight increase of Mn in step 2 with the adjusted extraction procedure; however, there was no difference whether 10 mL, 15 mL, or 20 mL of reagent was used.

Iron (Fe) was used to assess if the dissolution of hydrous and crystalline iron oxides happened due to the increased reagent to sample ratio in extraction step 2. However, Fe proved to be of limited use because its concentration in the samples was often below the detection limit in some of the extraction steps (Tables A.2–A.5, SI).

**Fig. 3:**
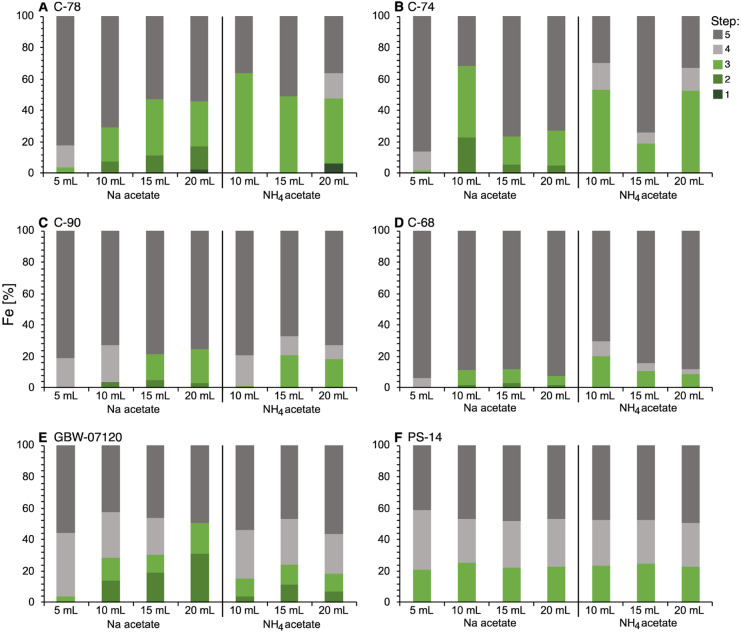
Fe (%) in extraction steps 1*–*5 for original (5 mL, NaOAc) and modified sequential extraction procedure (10, 15, and 20 mL, both NaOAc and NH_4_OAc) in all samples

The Posidonia shale sample (PS-14, Fig. 3F) had enough Fe extracted in steps 3 and 4 to allow detection and showed no Fe increase in extraction step 2 when increasing the reagent volume (Tables A.3–A.5, SI).

Molybdenum (Mo) was almost entirely dissolved in extraction step 1 (Tables A.2 and A.3, SI). In contrast, the As content in most samples was too low to be determined, except for sample C-68 (61 mg/kg), where As was detected mostly in extraction step 1 and to some smaller extent in extraction step 2 (Tables A.2 and A.3, SI). Furthermore, we observed no difference in Mo and As contents with respect to the reactant to sample ratio. The Posidonia shale (PS-14) had no measurable As, and the Mo content was 32 mg/kg, most of which was found in extraction step 4. For Mo and As, we did not observe any difference between extractions done with Na-acetate and NH_4_-acetate (Table A.3, SI).

## Discussion

### Adjustment of the reagent to sample ratio for carbonate extraction in CaCO_3_-rich samples

Calcium carbonate (CaCO_3_) calculated from Ca contents of the aqua regia digest agreed well with carbometer results and published values. Therefore, it was permissible to interpret Ca concentrations extracted in the individual extractions steps as representative for CaCO_3_. In sample C-74 (Fig. 1B), the CaCO_3_ concentration calculated from Ca was slightly higher (81 %) than the carbometer result (74 %), potentially resulting from the presence of Ca in mineral phases other than CaCO_3_. Since we observed complete removal of Ca in extraction step 2 in the optimized method (Table A.3, SI), the excess Ca had to be present in a carbonate mineral, such as dolomite, which was not detectable with the calcium carbometer. While the dissolution of calcite occurs very fast in the carbometer, the dissolution of dolomite is slower and takes approximately 15 min (De Blasio et al., 2013; Stumm, 1990). According to Loring (1976), the complete dissolution of CaCO_3_ is based on several factors such as the grain size, total carbonate content, and carbonate type. Since the samples in this study had the same grain size and similar carbonate content, only the carbonate type should have made a difference. In PS-14 (Fig. 1F), Ca extracted in extraction step 2 (resulting in 13 % CaCO_3_) agreed with the carbometer results (14 %), whereas the aqua regia-based bulk Ca overestimated the carbonate content (23 %). Actual extraction of Ca in step 1 suggested the presence of adsorbed Ca in addition to carbonate-bound Ca.

As seen in Fig. 1A–F, the extraction using the original sequential extraction procedure (Hall et al., 1996; Pichler et al., 2001) caused incomplete extraction of Ca in step 2 and substantial amounts of Ca in extraction steps 3 and 4. Similar results were observed by Davidson et al. (2006) during sequential extraction analyses of urban soils using the optimized BCR method (Rauret et al., 1999). They showed that in high-carbonate samples (> 30 % CaCO_3_), CaCO_3_ was not entirely dissolved by 0.11 mol L^-1^ acetic acid, resulting in a pH increase in the next extraction step, and thus impeding the extraction in the next step (Davidson et al., 2006).

Interestingly, in our study, the amount of Ca extracted in extraction step 2 was roughly the same (~ 100,000 mg/kg) for all CaCO_3_ samples (Table A.2, SI). Assuming the Ca in this to be entirely derived from CaCO_3_ resulted in approximately 26 to 27 weight-% CaCO_3_ extracted. In relatively low CaCO_3_ samples, such as sample C-68, this resulted in the dissolution of nearly half of the CaCO_3_ in extraction step 2. In contrast, in those samples with a higher CaCO_3_ content (> 80 %), only approximately one-third was extracted. That confirmed the limitation of the method and the need to optimize the original sequential extraction procedure to remove the entire CaCO_3_ in extraction step 2.

To improve extraction in step 2, we modified the reagent to sample ratio but kept the other steps (including step 1) unchanged. Based on the results from the original sequential extraction procedure, it was assumed that increasing the reagent would cause a more complete extraction of CaCO_3_ in step 2. While there was already a significant increase in extraction efficiency for Ca in step 2 using 10 mL and 15 mL of Na-acetate and NH_4_-acetate solution, we observed nearly complete removal of Ca in step 2 when adding 20 mL of the extracting reagent (Fig. 1A–F). This is supported by complete dissolution of pure calcium carbonate (Carl Roth, Germany) when using 20 mL extraction solution (not shown). Our results indicated that increasing the reagent to sample ratio by a factor of 4 compared to what has been suggested for standard samples was a good starting point for investigating the correct amount of reagent needed. Nevertheless, it seemed appropriate to carefully determine the proper amount of reagent required for the sample matrix under investigation. Another possible approach would have been a repetition of the carbonate removal step to dissolve all carbonates, as was done in a previous study (Sulkowski & Hirner, 2006). While this would have had the advantage of using more sediment with the available equipment (see section “Adjustments to the sequential extraction procedure”), we omitted this procedure to avoid possible sediment loss while decanting the extracting solution between the individual steps.

Manganese (Mn) was expected to be present as a minor element in CaCO_3_ or as rhodochrosite (MnCO_3_). Fig. 2A–F show that the extraction of Mn was not as systematic as the extraction of Ca. We observed a clear trend of increasing Ca concentration in step 2 when increasing the reagent volume, resulting in a complete carbonate removal in step 2 with 20 mL reagent. In contrast, some Mn was present in the extracts from step 3, particularly those with carbonate contents > 80 % (C-90, GBW). One reason might be the incomplete dissolution of Mn-carbonates in these samples. Another possibility could have been the presence of a small amount of other Mn phases, e.g., Mn oxides. Because in those two samples, the amount of Mn in step 3 decreased with an increasing amount of reagent, we rejected the latter and assumed incomplete removal of Mn carbonates. The results suggest that further increasing the reagent to samples ratio would result in complete Mn carbonate dissolution in these samples.

An important observation was that increasing the volume of reagent in step 2 did not compromise the subsequent extraction steps by attacking hydrous and crystalline Fe oxides or sulfides already in step 2. While most samples used in this study had relatively low Fe concentrations, the Posidonia shale sample (PS-14) demonstrated that a large reagent volume in step 2 did not cause larger amounts of Fe in extraction step 2 (Tables A.3–A.5, S1). In particular, the detailed study of sample PS-14, in which all reagent to sample ratios were covered, clearly showed no effect of the increased reagent to sample ratios on Fe concentrations (Fig. 3F). For Fe and Mg in the carbonate samples, it was noticeable that the sum did not match the aqua regia digestions exactly. We suspected that this was due to the relatively small amount of material used for the extractions (0.25 g), which likely caused sample inhomogeneity and an increased risk of sediment loss due to the transfer between extraction steps. We, therefore, recommend choosing a larger amount of sediment material and corresponding solvent amount if possible. However, a critical result from this study was that due to incomplete carbonate dissolution in step 2 using the original procedure, the extraction of hydrous and crystalline iron was inhibited, as seen in low Fe in steps 3 and 4 (Fig. 3A–E). The modified extraction procedure, which fully dissolved carbonates in step 2, allows the extraction of these phases in designated steps 3 and 4.

### Na-acetate vs. NH_4_-acetate extraction

**Fig. 4:**
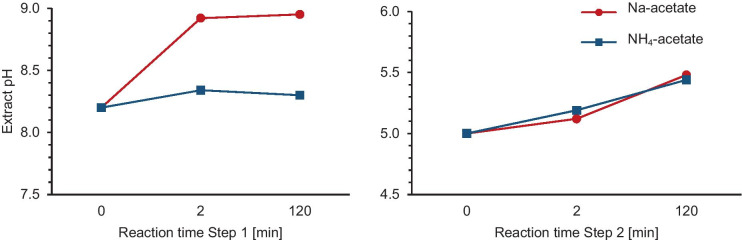
Changes in pH values in extraction steps 1 and 2 for pure CaCO_3_. The pH value was adjusted to 8.2 and 5.0, respectively, in the extraction solutions (0 min) measured after the addition to the extracts (2 min) and at the end of the reaction (120 min). Note that the x-axis is not on a linear scale

Both reagents, Na- and NH_4_-acetate, delivered a similar extraction efficiency for Ca and Mn in step 2. While Na-acetate, as well as NH_4_-acetate, was used extensively for the extraction of adsorbed/exchangeable and carbonate phases (e.g., Gleyzes et al., 2001; Tessier et al., 1979), the typical extractant for the carbonate phase was Na-acetate buffered to a pH of 5 (e.g., Gleyzes et al., 2001; Pickering, 1986). Because dissolution of carbonate is achieved by reaction with H^+^ (Gleyzes et al., 2002), it is not surprising that Na-acetate and NH_4_-acetate, both adjusted to a pH of 5, dissolve the same amounts of Ca (i.e., carbonate) in extraction step 2. Concerning extraction step 1, several studies showed that an NH_4_-acetate solution at a pH of 7.0 is a suitable solvent for releasing the adsorbed/exchangeable fraction, although it was suspected to react with the carbonate fraction (Chapman, 1965; Jackson, 1958; Tessier et al., 1979 & references therein; Wagemann et al., 1977). While this may explain higher Ca values in the first extraction step using NH_4_-acetate compared to Na-acetate, absolute concentrations of Ca in extraction step 1 and differences between Na- and NH_4_-acetate are too low to compromise the results for the high-carbonate samples used in this study (Table A.3, SI). However, to lower the potential for dissolution of CaCO_3_, we did not use NH_4_-acetate at a pH of 7, as discussed in Tessier et al. (1979), but adjusted the pH to 8.2 (as for Na-acetate). It was reported that the extraction of Ca from limestone starts at pH < 8.1 (Carrow & Duncan, 2011), which supported the use of a pH of 8.2. In summary, even if it was not possible to exclude any attack of carbonates by NH_4_-acetate entirely, there was no significant effect, which could compromise interpretations. Nevertheless, the use of NH_4_-acetate as a reagent in the first extraction step should be considered carefully for the sequential extraction of materials with less carbonate content. Monitoring the pH in pure CaCO_3_ (Carl Roth, Germany) during extraction steps 1 and 2 showed that the pH in the NH_4_-acetate solution increased only slightly in step 1. In contrast, it increased from 8.20 to 8.95 in Na-acetate (Fig. 4). That indicated that the system was buffered less in the Na-acetate solution in step 1.

**Fig. 5:**
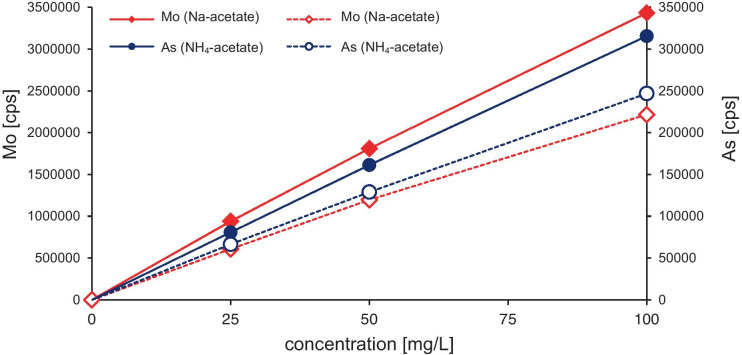
Intensities in counts per second (cps) for Mo and As during ICP-OES analysis in 0.33 M NH_4_-acetate and Na-acetate

While there was no advantage of NH_4_- over Na-acetate as a reagent during the extraction, the real benefit of using NH_4_-acetate became apparent during the chemical analyses of the extracts. It is well-known that in ICP-OES analyses, the presence of Na ions can suppress the analyte intensities of several elements (e.g., Brenner et al., 1999; Brenner et al., 1997; Ivaldi & Tyson, 1995). Accordingly, during our preliminary tests for the analysis of Mo and As, we observed approximately 20 % (As) and 35 % (Mo) higher intensities using 0.33 M NH_4_-acetate compared to 0.33 M Na-acetate (Fig. 5). Hence, the use of NH_4_-acetate in extractions steps 1 and 2 prevented signal suppression by Na. While signal intensities were not an issue for major elements such as Ca, Mg, and sometimes Fe or Mn, trace metals released during sequential extraction steps often had concentrations close to or below the limit of detection by ICP-OES. For these elements, signal enhancement by 20 to 35 % can be decisively important.

### Extraction efficiency for Mo and As

**Fig. 6:**
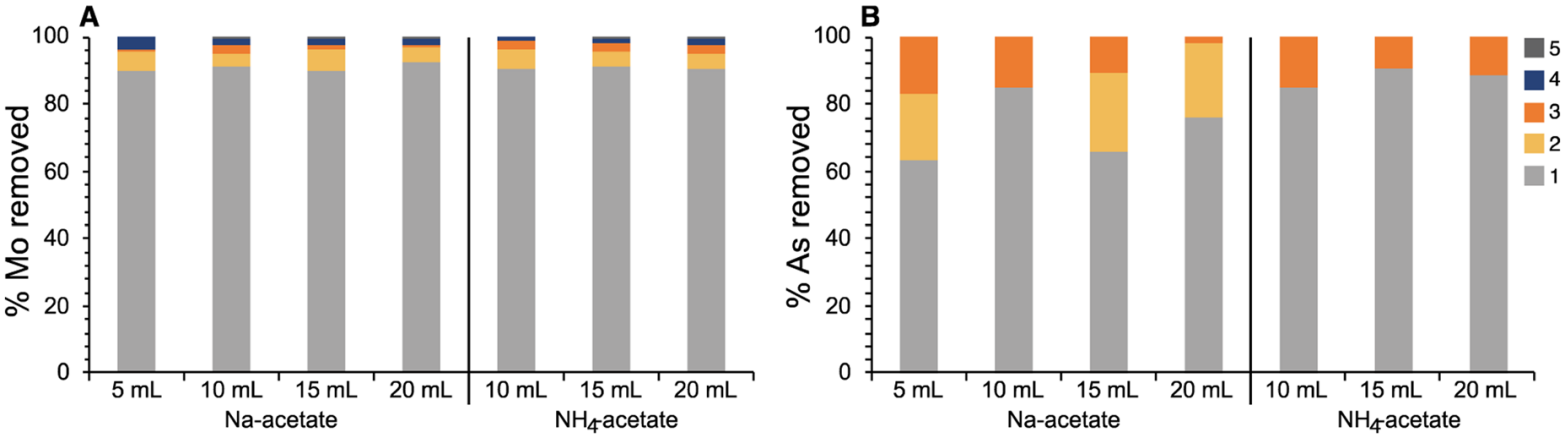
Portions of Mo and As removed in extraction steps 1*–*5 for the original (5 mL NaOAc) and modified sequential extraction procedure in sample C-68. As shows higher fluctuations than Mo due to low concentrations in steps 2*–*5

Most samples used in this study were from the Floridan Aquifer System where As and Mo are known contaminants (e.g., Lazareva & Pichler, 2007; Pichler & Mozaffari, 2015); and thus, both were included in the suite of analytes. Molybdenum and As were almost wholly extracted in step 1. While this was a systematic observation for Mo in the carbonate samples, we had only one sample with sufficient As, C-68. While the results were comparable, the use of NH_4_-acetate compared to Na-acetate produced stable concentrations for the complete extraction procedure regardless of the amount of reagent used in step 2 (Fig. 6). The larger variations for the extractions of As with Na-acetate in steps 2 and 3 were in part owed to the low concentrations, which favored the NH_4_-acetate extracts due to their analytical advantage (see above).

Molybdenum adsorbs as molybdate (MoO_4_^2-^) or thiomolybdate (MoO_n_S_4-n_^2-^) onto oxides (Fe, Mn, Al, Ti), pyrite, organic matter, and clay minerals, depending on the geochemical conditions (Bibak & Borggaard, 1994; Goldberg et al., 1996; Smedley & Kinniburgh, 2017; Xu et al., 2013). Arsenic adsorbs as arsenite (As^3+^) and arsenate (As^5+^) onto oxides (Fe, Mn, Al), clay minerals, and the surface of calcite (Smedley & Kinniburgh, 2002). The fact that both elements were observed to be mostly extracted in step 1 in the original extraction procedure suggested that the low reagent to sample ratio used in this approach was enough to release the adsorbed/exchangeable Mo and As from the solid phase. Those results agreed with what was found by Pichler and Mozaffari (2015).

**Fig. 7:**
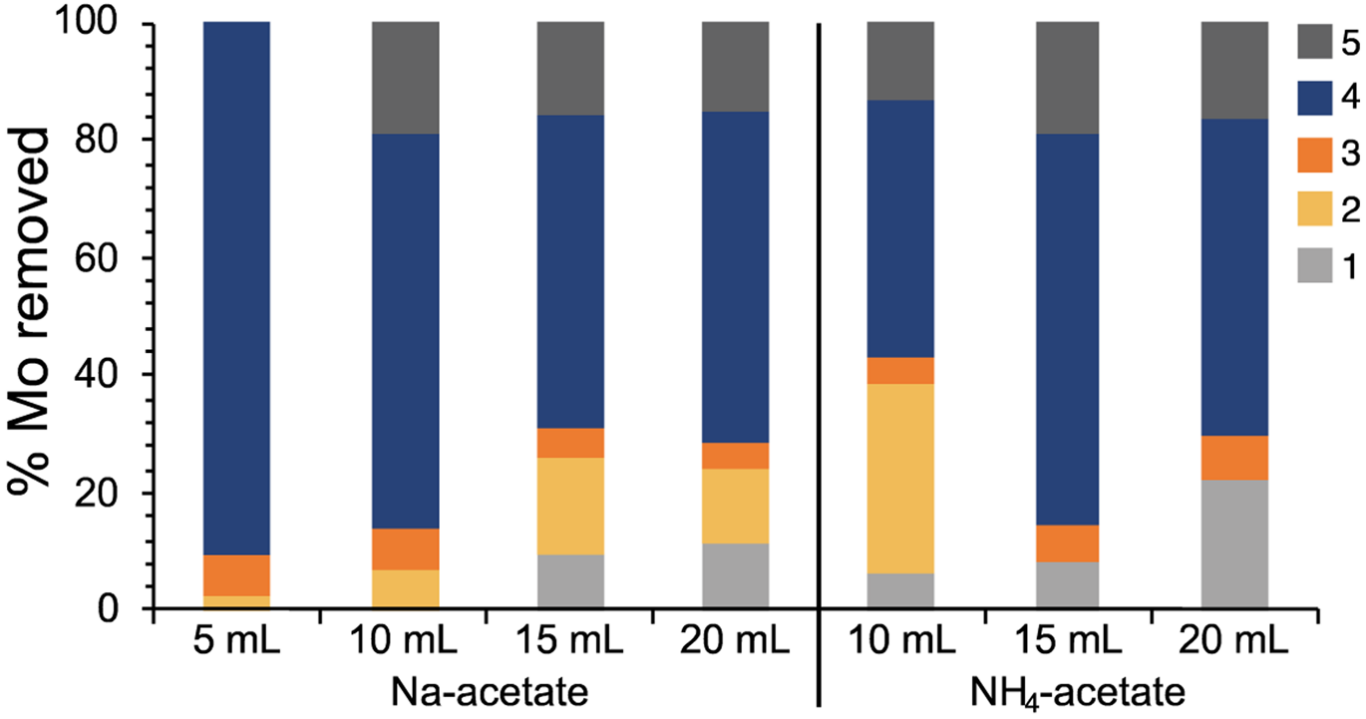
Portions of Mo removed in extraction steps 1 to 5 for the original (5 mL NaOAC) and modified sequential extraction procedures (remaining) in sample PS-14

In sample PS-14, which was not a carbonate sediment but had a CaCO_3_ of 14 %, Mo was mainly bound in the sulfide/organic matter fraction (Table A.3, SI; Fig. 7) as expected for TOC and pyrite-rich samples (e.g., Brumsack, 1991; Chappaz et al., 2014). An increase in the amount of reagent in step 2 did not affect the extraction of Mo contents in the following steps, an observation similar to what was seen for Fe (Fig. 3F). Thus, we concluded that the higher reagent to sample ratio did not affect Mo extraction, even in samples with low CaCO_3_ contents. That observation is of particular importance for using the same extraction procedure for extraction studies concerning samples with a wide range of CaCO_3_ concentrations.

## Conclusions

Our study demonstrated the need to adjust sequential extraction procedures to the research objectives and matrix requirements.

High-CaCO_3_ samples need more volume of reagent in the 2nd extraction step. Even with lower amounts of CaCO_3_ (see PS-14), the method is applicable. There is no need to adjust the method for samples with lower CaCO_3_ contents within a set of samples with different CaCO_3_ contents. An incomplete dissolution of CaCO_3_ in step 2 causes leftover CaCO_3_ in the subsequent steps, which prevents the dissolution of designated phases in these steps, namely hydrous and crystalline oxides. The adjusted protocol thus improves the extraction of oxide phases in the later extraction steps. A larger amount of extraction reagent in step 2 does not compromise following extractions steps, i.e., we observed no enhanced dissolution of Fe or Mo in step 2. NH_4_-Acetate can replace Na-acetate in extraction steps 1 and 2 to reduce Na interference in the plasma during ICP-OES measurements, which can be of particular importance for studying trace metals, such as As and Mo.

## Supplementary information

Below is the link to the electronic supplementary material.

Supplementary file1 (DOCX 122 KB)

## Data Availability

All data generated or analyzed during this study are included in this published article (and its supplementary information files).
