# Supplementary material for: Optimization and assessment of a sequential extraction procedure for calcium carbonate rocks
Source: Environ Monit Assess. 2021 Aug 16;193(9):577. doi: 10.1007/s10661-021-09300-x (PMC8364905; doi:10.1007/s10661-021-09300-x)
Supplement: Supplementary file 1 — Supplementary file1 (DOCX 122 KB) [file 10661_2021_9300_MOESM1_ESM.docx]

**Supplementary Online Material**

Table A.1. Elemental concentrations in aqua regia digests. Triplicates were only done for C-79, C-81, C-89, C-63, GBW-07120, PS-14. CaCO_3_ is calculated from measured Ca contents. Samples indicated by “n.a.” (not analyzed) have been lost during or prior to analysis. Data indicated by “n.d.” (not determined) are not reported due to a standard deviation > 10% during replicate detection (for details see text).

| **Sample** | **Ca** | **Mg** | **Sr** | **Fe** | **Mn** | **Mo** | **As** | **CaCO3** |
| --- | --- | --- | --- | --- | --- | --- | --- | --- |
| **Unit** | **mg/kg** | **mg/kg** | **mg/kg** | **mg/kg** | **mg/kg** | **mg/kg** | **mg/kg** | **%** |
|  |  |  |  |  |  |  |  |  |
| **C-78 a** | 313333 | 2758 | 311 | 950 | 20 | 49 | n.d. | 78 |
| **C-78 b** | 316976 | 2900 | 304 | 954 | 20 | 47 | n.d. | 79 |
| **C-78 c** | 313709 | 2737 | 310 | 937 | 21 | 44 | n.d. | 78 |
| **Average** | 314673 | 2798 | 308 | 947 | 20 | 47 |  | 79 |
| **Std. Dev.** | 2004 | 89 | 4 | 9 | 0.3 | 2 |  |  |
| **RSD (%)** | 0.6 | 3.2 | 1.2 | 1.0 | 1.4 | 5.3 |  |  |
|  |  |  |  |  |  |  |  |  |
| **C-74 a** | 324793 | 2702 | 298 | 925 | 25 | 36 | n.d. | 81 |
| **C-74 b** | 323249 | 2875 | 299 | 924 | 25 | 36 | n.d. | 81 |
| **C-74 c** | 329762 | 2983 | 303 | 965 | 25 | 36 | n.d. | 82 |
| **Average** | 325935 | 2853 | 300 | 938 | 25 | 36 |  | 81 |
| **Std. Dev.** | 3404 | 142 | 3 | 23 | 0.3 | 0.1 |  |  |
| **RSD (%)** | 1.0 | 5.0 | 1.0 | 2.5 | 1.3 | 0.4 |  |  |
|  |  |  |  |  |  |  |  |  |
| **C-90 a** | 342742 | 5541 | 1247 | 503 | 28 | 9 | n.d. | 86 |
| **C-90 b** | 359803 | 6162 | 1321 | 557 | 30 | 10 | n.d. | 90 |
| **C-90 c** | 368959 | 6152 | 1322 | 553 | 29 | 10 | n.d. | 92 |
| **Average** | 357168 | 5952 | 1297 | 538 | 29 | 10 |  | 89 |
| **Std. Dev.** | 13306 | 356 | 43 | 30 | 1 | 1 |  |  |
| **RSD (%)** | 3.7 | 6.0 | 3.3 | 5.6 | 3.4 | 6 |  |  |
|  |  |  |  |  |  |  |  |  |
| **C-68 a** | 240637 | 3038 | 438 | 1466 | 27 | 328 | 60 | 60 |
| **C-68 b** | 263465 | 2762 | 479 | 1249 | 25 | 322 | 60 | 66 |
| **C-68 c** | 253040 | 2971 | 463 | 1446 | 27 | 341 | 63 | 63 |
| **Average** | 252381 | 2924 | 460 | 1387 | 26 | 330 | 61 | 63 |
| **Std. Dev.** | 11428 | 144 | 20 | 120 | 1 | 10 | 2 |  |
| **RSD (%)** | 4.5 | 4.9 | 4.5 | 8.7 | 4.3 | 3.0 | 3 |  |
|  |  |  |  |  |  |  |  |  |
| **GBW-07120 a** | 364826 | 3085 | 92 | 1033 | 18 | n.d. | n.d. | 91 |
| **GBW-07120 b** | 371488 | 3134 | 93 | 1039 | 18 | n.d. | n.d. | 93 |
| **GBW-07120 c** | 369711 | 3010 | 90 | 1037 | 18 | n.d. | n.d. | 92 |
| **Average** | 368675 | 3076 | 92 | 1037 | 18 |  |  | 92 |
| **Std. Dev.** | 3450 | 63 | 1 | 3 | 0.1 |  |  |  |
| **RSD (%)** | 0.9 | 2.0 | 1.3 | 0.3 | 0.5 |  |  |  |
|  |  |  |  |  |  |  |  |  |
| **PS-14 a** | 91994 | 2768 | 483 | 30298 | 167 | 32 | n.d. | 23 |
| **PS-14 b** | 91151 | 2859 | 487 | 30435 | 168 | 31 | n.d. | 23 |
| **PS-14 c** | 94593 | 2889 | 492 | 30671 | 173 | 32 | n.d. | 24 |
| **Average** | 92579 | 2838 | 487 | 30468 | 169 | 32 |  | 23 |
| **Std. Dev.** | 1794 | 63 | 5 | 189 | 4 | 0.4 |  |  |
| **RSD (%)** | 1.9 | 2.2 | 1.0 | 0.6 | 2.1 | 1.2 |  |  |
|  |  |  |  |  |  |  |  |  |

Table A.2. Elemental concentrations in sequential extraction steps 1-5 using the original SEA. All extractions done in triplicate. *Sum of the average of triplicate extraction of each step. Samples indicated by “n.a.” (not analyzed) have been lost during or prior to analysis. Data indicated by “n.d.” (not determined) are not reported due to a standard deviation > 10% during replicate detection (for details see text).

| **Sample** | **extraction** | **replicate** | **Ca** | **Mg** | **Sr** | **Fe** | **Mn** | **Mo** | **As** |
| --- | --- | --- | --- | --- | --- | --- | --- | --- | --- |
| **Unit** | **step** |  | **mg/kg** | **mg/kg** | **mg/kg** | **mg/kg** | **mg/kg** | **mg/kg** | **mg/kg** |
|  |  |  |  |  |  |  |  |  |  |
| **C-78** | **1** | **1** | 1412 | 180 | 4 | n.d. | n.d. | 74 | n.d. |
|  |  | **2** | 1493 | 192 | 5 | n.d. | n.d. | 76 | n.d. |
|  |  | **3** | 1132 | 173 | 4 | n.d. | n.d. | 64 | n.d. |
|  |  | **average** | 1346 | 182 | 5 |  |  | 71 |  |
|  | **2** | **1** | 103433 | 533 | 113 | n.d. | 10 | 1 | n.d. |
|  |  | **2** | 104397 | 507 | 106 | n.d. | 9 | 1 | n.d. |
|  |  | **3** | 104440 | 536 | 116 | n.d. | 9 | 1 | n.d. |
|  |  | **average** | 104090 | 526 | 112 |  | 9 | 1 |  |
|  | **3** | **1** | 118903 | 504 | 109 | 36 | 7 | 1 | n.d. |
|  |  | **2** | 107395 | n.a. | n.a. | n.a. | n.a. | n.a. | n.a. |
|  |  | **3** | 103211 | 469 | 104 | 27 | 5 | n.d. | n.d. |
|  |  | **average** | 109836 | 486 | 106 | 31 | 6 | 1 |  |
|  | **4** | **1** | 67187 | 566 | 93 | 117 | 6 | 74 | n.d. |
|  |  | **2** | 67191 | n.a. | n.a. | n.a. | n.a. | n.a. | n.a. |
|  |  | **3** | 60078 | n.a. | n.a. | n.a. | n.a. | n.a. | n.a. |
|  |  | **average** | 64819 | 566 | 93 | 117 | 6 | 74 |  |
|  | **5** | **1** | n.d. | 826 | n.d. | 684 | 3 | n.d. | n.d. |
|  |  | **2** | n.d. | 1101 | n.d. | 869 | 4 | n.d. | n.d. |
|  |  | **3** | n.d. | 537 | n.d. | 471 | 3 | n.d. | n.d. |
|  |  | **average** |  | 821 |  | 674 | 3 |  |  |
|  | **sum** | **average*** | 280091 | 2581 | 315 | 822 | 25 | 147 |  |
|  | **aqua regia** | **average** | 314673 | 2798 | 308 | 947 | 20 | 47 |  |
|  |  |  |  |  |  |  |  |  |  |
| **C-74** | **1** | **1** | 1471 | 241 | 5 | n.d. | n.d. | 43 | n.d. |
|  |  | **2** | 1434 | 225 | 4 | n.d. | n.d. | 36 | n.d. |
|  |  | **3** | 1485 | 239 | 5 | n.d. | n.d. | 41 | n.d. |
|  |  | **average** | 1464 | 235 | 4 |  |  | 40 |  |
|  | **2** | **1** | 102469 | 601 | 102 | n.d. | 11 | n.d. | n.d. |
|  |  | **2** | 112245 | 561 | 92 | n.d. | 10 | n.d. | n.d. |
|  |  | **3** | 104288 | 585 | 101 | n.d. | 10 | n.d. | n.d. |
|  |  | **average** | 106334 | 582 | 98 |  | 10 |  |  |
|  | **3** | **1** | 109377 | 510 | 93 | 18 | 7 | n.d. | n.d. |
|  |  | **2** | 110292 | 508 | 90 | 19 | 7 | n.d. | n.d. |
|  |  | **3** | 109778 | 539 | 94 | 18 | 8 | n.d. | n.d. |
|  |  | **average** | 109816 | 519 | 92 | 18 | 7 |  |  |
|  | **4** | **1** | 51469 | 573 | 73 | 115 | 7 | n.d. | n.d. |
|  |  | **2** | 36733 | 589 | 59 | 132 | 7 | n.d. | n.d. |
|  |  | **3** | 42365 | n.a. | n.a. | n.a. | n.a. | n.a. | n.a. |
|  |  | **average** | 43522 | 581 | 66 | 124 | 7 |  |  |
|  | **5** | **1** | n.d. | 1473 | n.d. | 985 | 4 | n.d. | n.d. |
|  |  | **2** | n.d. | 1300 | n.d. | 870 | 4 | n.d. | n.d. |
|  |  | **3** | n.d. | 1036 | n.d. | 717 | 4 | n.d. | n.d. |
|  |  | **average** |  | 1270 |  | 857 | 4 |  |  |
|  | **sum** | **average*** | 261136 | 3187 | 261 | 999 | 29 | 40 |  |
|  | **aqua regia** | **average** | 325935 | 2853 | 300 | 938 | 25 | 36 |  |

|  |  |  |  |  |  |  |  |  |  |
| --- | --- | --- | --- | --- | --- | --- | --- | --- | --- |
| **C-90** | **1** | **1** | 1196 | 141 | 11 | n.d. | n.d. | 52 | n.d. |
|  |  | **2** | 1205 | 135 | 11 | n.d. | n.d. | 48 | 2 |
|  |  | **3** | 1232 | 140 | 11 | n.d. | n.d. | 56 | 1 |
|  |  | **average** | 1211 | 139 | 11 |  |  | 52 | 2 |
|  | **2** | **1** | 104003 | 657 | 412 | n.d. | 10 | 1 | n.d. |
|  |  | **2** | 102761 | 596 | 375 | n.d. | 8 | 1 | n.d. |
|  |  | **3** | 101253 | 639 | 390 | n.d. | 10 | 1 | n.d. |
|  |  | **average** | 102672 | 631 | 392 |  | 9 | 1 |  |
|  | **3** | **1** | 134892 | 655 | 397 | 2 | 7 | n.d. | n.d. |
|  |  | **2** | 136950 | 568 | 366 | 1 | 6 | n.d. | n.d. |
|  |  | **3** | 135198 | 659 | 397 | 3 | 9 | n.d. | n.d. |
|  |  | **average** | 135680 | 628 | 387 | 2 | 7 |  |  |
|  | **4** | **1** | 106621 | 1175 | 443 | 94 | 24 | n.d. | n.d. |
|  |  | **2** | 115551 | n.a. | n.a. | n.a. | n.a. | n.a. | n.a. |
|  |  | **3** | 103104 | n.a. | n.a. | n.a. | n.a. | n.a. | n.a. |
|  |  | **average** | 108425 | 1175 | 443 | 94 | 24 |  |  |
|  | **5** | **1** | n.d. | 2647 | n.d. | 418 | n.d. | n.d. | n.d. |
|  |  | **2** | n.d. | 2498 | n.d. | 403 | n.d. | n.d. | n.d. |
|  |  | **3** | n.d. | 2606 | n.d. | 414 | n.d. | n.d. | n.d. |
|  |  | **average** |  | 2584 |  | 412 |  |  |  |
|  | **sum** | **average*** | 347989 | 5156 | 1234 | 508 | 40 | 53 | 2 |
|  | **aqua regia** | **average** | 357168 | 5952 | 1297 | 538 | 29 | 10 |  |
|  |  |  |  |  |  |  |  |  |  |
| **C-68** | **1** | **1** | 1870 | 302 | 9 | n.d. | n.d. | 314 | 35 |
|  |  | **2** | 1965 | 297 | 9 | n.d. | n.d. | 317 | 34 |
|  |  | **3** | 1870 | 295 | 9 | n.d. | n.d. | 326 | 37 |
|  |  | **average** | 1902 | 298 | 9 |  |  | 319 | 35 |
|  | **2** | **1** | 120417 | 375 | 203 | n.d. | 8 | 17 | n.d. |
|  |  | **2** | 105386 | 357 | 188 | n.d. | 9 | 21 | 11 |
|  |  | **3** | 99866 | 377 | 202 | n.d. | 9 | 20 | n.d. |
|  |  | **average** | 108557 | 370 | 197 |  | 9 | 19 | 11 |
|  | **3** | **1** | 92359 | 138 | 146 | n.d. | 5 | 1 | 10 |
|  |  | **2** | 95924 | 182 | 150 | n.d. | 5 | 2 | 10 |
|  |  | **3** | 101862 | 176 | 154 | n.d. | 5 | 3 | 9 |
|  |  | **average** | 96715 | 165 | 150 |  | 5 | 2 | 10 |
|  | **4** | **1** | 1250 | 452 | n.d. | n.d. | 2 | 13 | n.d. |
|  |  | **2** | 2130 | 460 | 6 | 94 | 3 | 15 | n.d. |
|  |  | **3** | 6940 | 482 | 16 | 79 | 3 | 16 | n.d. |
|  |  | **average** | 3440 | 464 | 11 | 87 | 3 | 15 |  |
|  | **5** | **1** | n.d. | 1827 | n.d. | 1538 | 17 | n.d. | n.d. |
|  |  | **2** | n.d. | 1169 | n.d. | 1031 | 12 | n.d. | n.d. |
|  |  | **3** | 88 | 1656 | n.d. | 1405 | 15 | n.d. | n.d. |
|  |  | **average** | 88 | 1551 |  | 1325 | 14 |  |  |
|  | **sum** | **average*** | 210701 | 2848 | 368 | 1411 | 31 | 355 | 56 |
|  | **aqua regia** | **average** | 252381 | 2924 | 460 | 1387 | 26 | 330 | 61 |

|  |  |  |  |  |  |  |  |  |  |
| --- | --- | --- | --- | --- | --- | --- | --- | --- | --- |
| **GBW-07120** | **1** | **1** | 1202 | 64 | 2 | n.d. | n.d. | n.d. | n.d. |
|  |  | **2** | 887 | 64 | 2 | n.d. | n.d. | n.d. | n.d. |
|  |  | **3** | 1109 | 63 | 2 | n.d. | n.d. | n.d. | n.d. |
|  |  | **average** | 1066 | 64 | 2 |  |  |  |  |
|  | **2** | **1** | 103573 | n.a. | n.a. | n.a. | n.a. | n.a. | n.a. |
|  |  | **2** | 113784 | 418 | 28 | 4 | 6 | n.d. | n.d. |
|  |  | **3** | 104632 | 408 | 27 | 2 | 6 | n.d. | n.d. |
|  |  | **average** | 107330 | 413 | 28 | 3 | 6 |  |  |
|  | **3** | **1** | 145496 | n.a. | n.a. | n.a. | n.a. | n.a. | n.a. |
|  |  | **2** | 146753 | 400 | 30 | 35 | 7 | n.d. | n.d. |
|  |  | **3** | 139906 | n.a. | n.a. | n.a. | n.a. | n.a. | n.a. |
|  |  | **average** | 144052 | 400 | 30 | 35 | 7 |  |  |
|  | **4** | **1** | 88748 | 774 | 30 | 391 | 8 | n.d. | n.d. |
|  |  | **2** | 88885 | 796 | 31 | 395 | 8 | n.d. | n.d. |
|  |  | **3** | 94531 | n.a. | n.a. | n.a. | n.a. | n.a. | n.a. |
|  |  | **average** | 90721 | 785 | 31 | 393 | 8 |  |  |
|  | **5** | **1** | n.d. | 795 | n.d. | 510 | n.d. | n.d. | n.d. |
|  |  | **2** | 67 | 923 | n.d. | 573 | n.d. | n.d. | n.d. |
|  |  | **3** | n.d. | 955 | n.d. | 566 | n.d. | n.d. | n.d. |
|  |  | **average** | 67 | 891 |  | 549 |  |  |  |
|  | **sum** | **average*** | 343235 | 2552 | 90 | 980 | 21 |  |  |
|  | **aqua regia** | **average** | 368675 | 3076 | 92 | 1037 | 18 |  |  |
|  |  |  |  |  |  |  |  |  |  |
| **PS-14** | **1** | **1** | 21662 | 89 | 94 | n.d. | 0.2 | n.d. | n.d. |
|  |  | **2** | 20042 | n.a. | n.a. | n.a. | n.a. | n.a. | n.a. |
|  |  | **3** | 20844 | 85 | 83 | n.d. | 0.1 | n.d. | n.d. |
|  |  | **average** | 20849 | 87 | 89 |  | 0.2 |  |  |
|  | **2** | **1** | 54677 | 392 | 340 | 29 | 56 | 1 | n.d. |
|  |  | **2** | 52475 | 442 | 330 | 30 | 60 | n.d. | n.d. |
|  |  | **3** | 52469 | 428 | 344 | 29 | 59 | n.d. | n.d. |
|  |  | **average** | 53207 | 421 | 338 | 29 | 58 | 1 |  |
|  | **3** | **1** | 6313 | 821 | 29 | 6683 | 37 | 3 | n.d. |
|  |  | **2** | 6736 | 846 | 30 | 5699 | 33 | 2 | n.d. |
|  |  | **3** | 6088 | 896 | 31 | 7189 | 3 | 3 | n.d. |
|  |  | **average** | 6379 | 855 | 30 | 6524 | 25 | 3 |  |
|  | **4** | **1** | 284 | n.a. | n.a. | n.a. | n.a. | n.a. | n.a. |
|  |  | **2** | 432 | 93 | 3 | 11999 | 63 | 38 | 14 |
|  |  | **3** | 221 | n.a. | n.a. | n.a. | n.a. | n.a. | n.a. |
|  |  | **average** | 312 | 93 | 3 | 11999 | 63 | 38 | 14 |
|  | **5** | **1** | 121 | 687 | n.d. | 13396 | 20 | n.d. | n.d. |
|  |  | **2** | 158 | 667 | n.d. | 13006 | 20 | n.d. | n.d. |
|  |  | **3** | 110 | 687 | n.d. | 12840 | 20 | n.d. | n.d. |
|  |  | **average** | 130 | 680 |  | 13081 | 20 |  |  |
|  | **sum** | **average*** | 80877 | 2135 | 460 | 31633 | 166 | 42 | 14 |
|  | **aqua regia** | **average** | 92579 | 2838 | 487 | 30468 | 169 | 32 |  |

Table A.3. Elemental concentrations in sequential extraction steps 1-5 using the modified SEA (20 mL Na-acetate and NH_4_-acetate. respectively). Samples indicated by “n.a.” (not analyzed) have been lost during or prior to analysis. Data indicated by “n.d.” (not determined) are not reported due to a standard deviation > 10% during replicate detection (for details see text).

| **Sample** | **solution** | **extraction** | **Ca** | **Mg** | **Sr** | **Fe** | **Mn** | **Mo** | **As** |
| --- | --- | --- | --- | --- | --- | --- | --- | --- | --- |
| **Unit** |  | **step** | **mg/kg** | **mg/kg** | **mg/kg** | **mg/kg** | **mg/kg** | **mg/kg** | **mg/kg** |
|  |  |  |  |  |  |  |  |  |  |
| **C-78** | **Na-acetate** | **1** | 1543 | 122 | 5 | 7 | n.d. | 52 | n.d. |
|  |  | **2** | 340965 | 1308 | 282 | 37 | 23 | 7 | n.d. |
|  |  | **3** | 1728 | 131 | 7 | 72 | 0.2 | n.d. | 0.3 |
|  |  | **4** | 331 | 79 | 1 | 24 | 1 | n.d. | n.a. |
|  |  | **5** | n.d. | 131 | n.d. | 135 | 0.5 | n.d. | n.d. |
|  |  | **sum** | 344567 | 1771 | 295 | 275 | 25 | 59 | 0.3 |
|  | **NH4-acetate** | **1** | 4197 | 123 | 6 | n.d. | n.d. | 54 | n.d. |
|  |  | **2** | 287773 | 973 | 252 | n.d. | 15 | n.d. | n.d. |
|  |  | **3** | 989 | 90 | 5 | 47 | n.d. | n.d. | n.d. |
|  |  | **4** | n.d. | 56 | n.d. | 18 | n.d. | n.d. | n.d. |
|  |  | **5** | n.d. | n.d. | n.d. | 41 | n.d. | n.d. | n.d. |
|  |  | **sum** | 292959 | 1242 | 263 | 105 | 15 | 54 |  |
|  | **aqua regia** | **average** | 314673 | 2798 | 308 | 947 | 20 | 47 |  |
|  |  |  |  |  |  |  |  |  |  |
| **C-74** | **Na-acetate** | **1** | 1626 | 139 | 5 | 7 | n.d. | 46 | n.d. |
|  |  | **2** | 333077 | 1365 | 257 | 21 | 24 | 4 | n.d. |
|  |  | **3** | 2834 | 154 | 10 | 87 | 0.4 | n.d. | n.d. |
|  |  | **4** | n.a. | n.a. | n.a. | n.a. | n.a. | n.a. | n.a. |
|  |  | **5** | n.d. | 314 | n.d. | 295 | 1 | n.d. | n.d. |
|  |  | **sum** | 337537 | 1973 | 272 | 410 | 25 | 50 |  |
|  | **NH4-acetate** | **1** | 4387 | 143 | 6 | n.d. | n.d. | 43 | n.d. |
|  |  | **2** | 380629 | 1234 | 289 | n.d. | 24 | n.d. | n.d. |
|  |  | **3** | 2915 | 164 | 11 | 85 | 0.5 | n.d. | n.d. |
|  |  | **4** | n.d. | 78 | n.d. | 23 | n.d. | n.d. | n.d. |
|  |  | **5** | n.d. | n.d. | n.d. | 53 | n.d. | n.d. | n.d. |
|  |  | **sum** | 387932 | 1620 | 306 | 162 | 24 | 43 |  |
|  | **aqua regia** | **average** | 325935 | 2853 | 300 | 938 | 25 | 36 |  |
|  |  |  |  |  |  |  |  |  |  |
| **C-90** | **Na-acetate** | **1** | 1525 | 85 | 13 | n.d. | n.d. | 12 | n.d. |
|  |  | **2** | 415071 | 1966 | 1241 | 12 | 31 | 4 | n.d. |
|  |  | **3** | 406 | 478 | 2 | 84 | 3 | n.d. | n.d. |
|  |  | **4** | n.d. | 472 | n.d. | n.d. | n.d. | n.d. | n.d. |
|  |  | **5** | n.d. | 1626 | n.d. | 290 | n.d. | n.d. | n.d. |
|  |  | **sum** | 417003 | 4627 | 1255 | 386 | 34 | 16 |  |
|  | **NH4-acetate** | **1** | 4615 | 131 | 19 | n.d. | n.d. | 12 | n.d. |
|  |  | **2** | 389131 | 1644 | 1322 | n.d. | 30 | n.d. | n.d. |
|  |  | **3** | n.d. | 469 | 2 | 76 | 3 | n.d. | n.d. |
|  |  | **4** | 292 | 461 | n.d. | 34 | n.d. | n.d. | n.d. |
|  |  | **5** | n.d. | 1661 | n.d. | 297 | n.d. | n.d. | n.d. |
|  |  | **sum** | 394039 | 4366 | 1343 | 407 | 33 | 12 |  |
|  | **aqua regia** | **average** | 357168 | 5952 | 1297 | 538 | 29 | 10 |  |
|  |  |  |  |  |  |  |  |  |  |
| **C-68** | **Na-acetate** | **1** | 2175 | 208 | 9 | n.d. | n.d. | 327 | 37 |
|  |  | **2** | 205803 | 602 | 383 | 19 | 13 | 17 | 11 |
|  |  | **3** | 1401 | 68 | 3 | 51 | n.d. | 2 | 1 |
|  |  | **4** | n.d. | n.d. | n.d. | n.d. | n.d. | 6 | n.d. |
|  |  | **5** | n.d. | 911 | n.d. | 850 | 8 | 4 | n.d. |
|  |  | **sum** | 209379 | 1789 | 395 | 921 | 22 | 356 | 49 |
|  | **NH4-acetate** | **1** | 4495 | 235 | 13 | n.d. | n.d. | 371 | 39 |
|  |  | **2** | 283447 | 463 | 418 | n.d. | 12 | 20 | n.d. |
|  |  | **3** | 984 | 115 | 5 | 58 | 0.4 | 10 | 5 |
|  |  | **4** | n.d. | 99 | n.d. | 22 | n.d. | 7 | n.d. |
|  |  | **5** | n.d. | 626 | n.d. | 583 | 6 | 4 | n.d. |
|  |  | **sum** | 288926 | 1538 | 435 | 662 | 18 | 412 | 44 |
|  | **aqua regia** | **average** | 252381 | 2924 | 460 | 1387 | 26 | 330 | 61 |

|  |  |  |  |  |  |  |  |  |  |
| --- | --- | --- | --- | --- | --- | --- | --- | --- | --- |
| **GBW-07120** | **Na-acetate** | **1** | 1353 | 13 | n.d. | n.d. | n.d. | 1 | n.d. |
|  |  | **2** | 325464 | 1178 | 95 | 73 | 16 | 6 | n.d. |
|  |  | **3** | 378 | 194 | 0.3 | 46 | 1 | n.d. | n.d. |
|  |  | **4** | n.d. | n.d. | 0.4 | n.d. | n.d. | n.d. | n.d. |
|  |  | **5** | n.d. | n.d. | n.d. | 115 | n.d. | n.d. | 1 |
|  |  | **sum** | 327195 | 1386 | 96 | 233 | 17 | 7 | 1 |
|  | **NH4-acetate** | **1** | 4532 | 37 | 3 | n.d. | n.d. | n.d. | n.d. |
|  |  | **2** | 350662 | 1113 | 105 | 36 | 17 | 6 | n.d. |
|  |  | **3** | 409 | 233 | 0.3 | 58 | 2 | n.d. | n.d. |
|  |  | **4** | n.d. | 117 | n.d. | 127 | n.d. | n.d. | n.d. |
|  |  | **5** | n.d. | 102 | n.d. | 285 | n.d. | n.d. | 2 |
|  |  | **sum** | 355602 | 1603 | 109 | 506 | 19 | 6 | 2 |
|  | **aqua regia** | **average** | 368675 | 3076 | 92 | 1037 | 18 |  |  |
|  |  |  |  |  |  |  |  |  |  |
| **PS-14** | **Na-acetate** | **1** | 16008 | 23 | 65 | n.d. | 1 | 3 | n.d. |
|  |  | **2** | 58194 | 1145 | 309 | 80 | 70 | 4 | n.d. |
|  |  | **3** | 3941 | 217 | 20 | 4382 | 17 | 1 | n.d. |
|  |  | **4** | n.d. | n.d. | n.d. | 6054 | 32 | 16 | 3 |
|  |  | **5** | 234 | 331 | 2 | 9198 | 15 | 4 | 1 |
|  |  | **sum** | 78377 | 1716 | 396 | 19713 | 136 | 29 | 5 |
|  | **NH4-acetate** | **1** | 20257 | 11 | 73 | n.d. | n.d. | 8 | n.d. |
|  |  | **2** | 52750 | 1079 | 303 | 22 | 67 | n.d. | n.d. |
|  |  | **3** | 3867 | 149 | 24 | 5368 | 20 | 3 | 1 |
|  |  | **4** | n.d. | 56 | 2 | 6697 | 37 | 21 | 4 |
|  |  | **5** | 278 | 442 | 3 | 11830 | 20 | 6 | n.d. |
|  |  | **sum** | 77153 | 1737 | 405 | 23917 | 144 | 39 | 5 |
|  | **aqua regia** | **average** | 92579 | 2838 | 487 | 30468 | 169 | 32 |  |

Table A.4. Elemental concentrations in sequential extraction steps 1-5 using the modified SEA (10 mL Na-acetate and NH_4_-acetate, respectively). Samples indicated by “n.a.” (not analyzed) have been lost during or prior to analysis. Data indicated by “n.d.” (not determined) are not reported due to a standard deviation > 10% during replicate detection (for details see text).

| **Sample** | **solution** | **extraction** | **Ca** | **Mg** | **Sr** | **Fe** | **Mn** | **Mo** | **As** |
| --- | --- | --- | --- | --- | --- | --- | --- | --- | --- |
| **Unit** |  | **step** | **mg/kg** | **mg/kg** | **mg/kg** | **mg/kg** | **mg/kg** | **mg/kg** | **mg/kg** |
|  |  |  |  |  |  |  |  |  |  |
| **C-78** | **Na-acetate** | **1** | 1721 | 119 | 5 | n.d. | n.d. | 56 | n.d. |
|  |  | **2** | 216014 | 1006 | 203 | 23 | 17 | 4 | n.d. |
|  |  | **3** | 61427 | 365 | 62 | 64 | 2.5 | n.d. | n.d. |
|  |  | **4** | 561 | n.d. | n.d. | n.d. | n.d. | n.d. | n.d. |
|  |  | **5** | n.d. | 230 | n.d. | 211 | 1.6 | n.d. | n.d. |
|  |  | **sum** | 279723 | 1720 | 271 | 298 | 22 | 60 |  |
|  | **NH4-acetate** | **1** | 3916 | 119 | 6 | n.d. | n.d. | 52 | n.d. |
|  |  | **2** | 196822 | 939 | 217 | n.d. | 15 | n.d. | n.d. |
|  |  | **3** | 20953 | 180 | 25 | 56 | 1 | 1 | n.d. |
|  |  | **4** | n.d. | n.d. | n.d. | n.d. | n.d. | n.d. | n.d. |
|  |  | **5** | n.d. | n.d. | n.d. | 32 | n.d. | n.d. | n.d. |
|  |  | **sum** | 221691 | 1238 | 248 | 89 | 16 | 53 | n.d. |
|  | **aqua regia** | **average** | 314673 | 2798 | 308 | 947 | 20 | 47 |  |
|  |  |  |  |  |  |  |  |  |  |
| **C-74** | **Na-acetate** | **1** | 1612 | 128 | 5 | n.d. | n.d. | 45 | n.d. |
|  |  | **2** | 229857 | 1032 | 198 | 16 | 20 | 2 | n.d. |
|  |  | **3** | 45629 | 238 | 41 | 32 | 2 | n.d. | n.d. |
|  |  | **4** | 385 | n.d. | n.d. | n.d. | n.d. | n.d. | n.d. |
|  |  | **5** | n.d. | n.d. | n.d. | 23 | n.d. | n.d. | n.d. |
|  |  | **sum** | 277484 | 1397 | 244 | 71 | 23 | 47 |  |
|  | **NH4-acetate** | **1** | 4435 | 167 | 6 | n.d. | n.d. | 53 | 4 |
|  |  | **2** | 271553 | 962 | 214 | n.d. | 20 | n.d. | n.d. |
|  |  | **3** | 33948 | 281 | 38 | 69 | 2 | n.d. | n.d. |
|  |  | **4** | n.d. | 89 | n.d. | 23 | n.d. | n.d. | n.d. |
|  |  | **5** | n.d. | n.d. | n.d. | 38 | n.d. | n.d. | n.d. |
|  |  | **sum** | 309936 | 1499 | 258 | 129 | 22 | 53 | 4 |
|  | **aqua regia** | **average** | 325935 | 2853 | 300 | 938 | 25 | 36 |  |
|  |  |  |  |  |  |  |  |  |  |
| **C-90** | **Na-acetate** | **1** | 1463 | 79 | 12 | n.d. | n.d. | 11 | n.d. |
|  |  | **2** | 201628 | 1173 | 782 | 14 | 22 | 3 | n.d. |
|  |  | **3** | 105331 | 563 | 306 | n.d. | 5 | n.d. | n.d. |
|  |  | **4** | n.d. | 649 | n.d. | 84 | 5 | n.d. | n.d. |
|  |  | **5** | n.d. | 1502 | n.d. | 263 | n.d. | n.d. | n.d. |
|  |  | **sum** | 308422 | 3967 | 1101 | 361 | 31 | 14 |  |
|  | **NH4-acetate** | **1** | 4636 | 127 | 19 | n.d. | n.d. | 12 |  |
|  |  | **2** | 247515 | 1155 | 901 | n.d. | 22 | 3 | n.d. |
|  |  | **3** | 94611 | 558 | 293 | 7 | 5 | n.d. | n.d. |
|  |  | **4** | 507 | 738 | n.d. | 90 | 5 | n.d. | n.d. |
|  |  | **5** | n.d. | 2187 | n.d. | 373 | n.d. | n.d. | n.d. |
|  |  | **sum** | 347269 | 4765 | 1214 | 469 | 32 | 15 |  |
|  | **aqua regia** | **average** | 357168 | 5952 | 1297 | 538 | 29 | 10 |  |
|  |  |  |  |  |  |  |  |  |  |
| **C-68** | **Na-acetate** | **1** | 2124 | 223 | 10 | n.d. | n.d. | 352 | 40 |
|  |  | **2** | 218600 | 661 | 393 | 15 | 16 | 15 | n.d. |
|  |  | **3** | 14765 | 167 | 28 | 69 | 1 | 10 | 7 |
|  |  | **4** | n.d. | n.d. | n.d. | n.d. | n.d. | 7 | n.d. |
|  |  | **5** | n.d. | 688 | n.d. | 656 | 6 | 4 | n.d. |
|  |  | **sum** | 235489 | 1739 | 431 | 739 | 23 | 389 | 47 |
|  | **NH4-acetate** | **1** | 4573 | 227 | 12 | n.d. | n.d. | 335 | 35 |
|  |  | **2** | 248840 | 607 | 419 | n.d. | 15 | 20 | n.d. |
|  |  | **3** | 9689 | 126 | 20 | 46 | 1 | 11 | 6 |
|  |  | **4** | n.d. | 61 | n.d. | 21 | n.d. | 5 | n.d. |
|  |  | **5** | n.d. | 106 | n.d. | 159 | 2 | n.d. | n.d. |
|  |  | **sum** | 263103 | 1126 | 451 | 226 | 18 | 370 | 42 |
|  | **aqua regia** | **average** | 252381 | 2924 | 460 | 1387 | 26 | 330 | 61 |

|  |  |  |  |  |  |  |  |  |  |
| --- | --- | --- | --- | --- | --- | --- | --- | --- | --- |
| **GBW-07120** | **Na-acetate** | **1** | 1344 | 33 | 4 | n.d. | n.d. | n.d. | n.d. |
|  |  | **2** | 246690 | 766 | 63 | 43 | 12 | 2 | 8 |
|  |  | **3** | 74073 | 394 | 19 | 45 | 3 | n.d. | n.d. |
|  |  | **4** | n.d. | n.d. | n.d. | 90 | n.d. | n.d. | n.d. |
|  |  | **5** | n.d. | n.d. | n.d. | 131 | n.d. | n.d. | n.d. |
|  |  | **sum** | 322108 | 1194 | 86 | 309 | 16 | 2 | 8 |
|  | **NH4-acetate** | **1** | 4180 | 34 | 3 | n.d. | n.d. | 1 | n.d. |
|  |  | **2** | 237584 | 684 | 59 | 16 | 11 | 4 | n.d. |
|  |  | **3** | 79139 | 432 | 20 | 46 | 4 | n.d. | n.d. |
|  |  | **4** | n.d. | 151 | n.d. | 122 | n.d. | n.d. | n.d. |
|  |  | **5** | n.d. | 129 | n.d. | 215 | n.d. | n.d. | n.d. |
|  |  | **sum** | 320903 | 1430 | 83 | 398 | 15 | 5 |  |
|  | **aqua regia** | **average** | 368675 | 3076 | 92 | 1037 | 18 | 1 |  |
|  |  |  |  |  |  |  |  |  |  |
| **PS-14** | **Na-acetate** | **1** | 12663 | n.a. | n.a. | n.a. | n.a. | n.a. | n.a. |
|  |  | **2** | 43939 | 823 | 253 | 65 | 56 | 2 | n.d. |
|  |  | **3** | 3792 | 266 | 20 | 4462 | 18 | 2 | n.d. |
|  |  | **4** | n.d. | n.d. | n.d. | 5095 | 25 | 15 | n.d. |
|  |  | **5** | 317 | 299 | 2 | 8383 | 14 | 4 | n.d. |
|  |  | **sum** | 60711 | 1387 | 276 | 18005 | 114 | 22 | 0.5 |
|  | **NH4-acetate** | **1** | 21253 | 44 | 83 | n.d. | 0.3 | 3 | n.d. |
|  |  | **2** | 88747 | 1089 | 310 | 39 | 65 | 15 | n.d. |
|  |  | **3** | 4617 | 251 | 24 | 5612 | 23 | 2 | 1 |
|  |  | **4** | n.d. | 61 | 2 | 7221 | 38 | 20 | n.d. |
|  |  | **5** | 302 | 379 | 3 | 11581 | 19 | 6 | n.d. |
|  |  | **sum** | 114919 | 1825 | 422 | 24453 | 145 | 45 | 1 |
|  | **aqua regia** | **average** | 92579 | 2838 | 487 | 30468 | 169 | 32 |  |
|  |  |  |  |  |  |  |  |  |  |

Table A.5. Elemental concentrations in sequential extraction steps 1-5 using the modified SEA (15 mL Na-acetate and NH_4_-acetate, respectively). Samples indicated by “n.a.” (not analyzed) have been lost during or prior to analysis. Data indicated by “n.d.” (not determined) are not reported due to a standard deviation > 10% during replicate detection (for details see text).

| **Sample** | **solution** | **extraction** | **Ca** | **Mg** | **Sr** | **Fe** | **Mn** | **Mo** | **As** |
| --- | --- | --- | --- | --- | --- | --- | --- | --- | --- |
| **Unit** |  | **step** | **mg/kg** | **mg/kg** | **mg/kg** | **mg/kg** | **mg/kg** | **mg/kg** | **mg/kg** |
|  |  |  |  |  |  |  |  |  |  |
| **C-78** | **Na-acetate** | **1** | 1674 | 113 | 5 | n.d. | n.d. | 57 | n.d. |
|  |  | **2** | 309374 | 1403 | 298 | 27 | 22 | 5 | n.d. |
|  |  | **3** | 3179 | 150 | 10 | 80 | 0.3 | 0.2 | n.d. |
|  |  | **4** | n.d. | n.d. | n.d. | n.d. | n.d. | n.d. | n.d. |
|  |  | **5** | n.d. | 122 | n.d. | 121 | n.d. | n.d. | n.d. |
|  |  | **sum** | 314227 | 1788 | 313 | 228 | 22 | 62 |  |
|  | **NH4-acetate** | **1** | 4286 | 124 | 6 | n.d. | n.d. | 54 | n.d. |
|  |  | **2** | 304058 | 1022 | 255 | n.d. | 16 | n.d. | n.d. |
|  |  | **3** | 1040 | 51 | 3 | 29 | n.d. | n.d. | n.d. |
|  |  | **4** | n.d. | n.d. | n.d. | n.d. | n.d. | n.d. | n.d. |
|  |  | **5** | n.d. | n.d. | n.d. | 30 | n.d. | n.d. | n.d. |
|  |  | **sum** | 309384 | 1198 | 264 | 60 | 16 | 54 | n.d. |
|  | **aqua regia** | **average** | 314673 | 2798 | 308 | 947 | 20 | 47 |  |
|  |  |  |  |  |  |  |  |  |  |
| **C-74** | **Na-acetate** | **1** | 1626 | 131 | 5 | n.d. | n.d. | 45 | n.d. |
|  |  | **2** | 280325 | 1374 | 267 | 29 | 26 | 5 | n.d. |
|  |  | **3** | 30167 | 276 | 32 | 88 | 2 | n.d. | n.d. |
|  |  | **4** | n.d. | n.d. | n.d. | n.d. | n.d. | n.d. | n.d. |
|  |  | **5** | n.d. | n.d. | n.d. | 383 | n.d. | n.d. | n.d. |
|  |  | **sum** | 312118 | 1781 | 304 | 500 | 28 | 51 |  |
|  | **NH4-acetate** | **1** | 3962 | 138 | 6 | n.d. | n.d. | 43 | n.d. |
|  |  | **2** | 329096 | 1226 | 275 | n.d. | 24 | n.d. | n.d. |
|  |  | **3** | 3555 | 142 | 10 | 74 | n.d. | n.d. | n.d. |
|  |  | **4** | n.d. | 94 | n.d. | 28 | n.d. | n.d. | n.d. |
|  |  | **5** | n.d. | 307 | n.d. | 296 | n.d. | n.d. | n.d. |
|  |  | **sum** | 336614 | 1906 | 291 | 398 | 24 | 43 |  |
|  | **aqua regia** | **average** | 325935 | 2853 | 300 | 938 | 25 | 36 |  |
|  |  |  |  |  |  |  |  |  |  |
| **C-90** | **Na-acetate** | **1** | 1483 | 80 | 12 | n.d. | n.d. | 11 | n.d. |
|  |  | **2** | 317140 | 1737 | 1146 | 21 | 31 | 4 | n.d. |
|  |  | **3** | 35536 | 594 | 109 | 70 | 5 | n.d. | n.d. |
|  |  | **4** | n.d. | 523 | n.d. | n.d. | n.d. | n.d. | n.d. |
|  |  | **5** | n.d. | 1857 | n.d. | 331 | n.d. | n.d. | n.d. |
|  |  | **sum** | 354160 | 4792 | 1267 | 422 | 36 | 16 |  |
|  | **NH4-acetate** | **1** | 4360 | 127 | 20 | n.d. | n.d. | 12 | n.d. |
|  |  | **2** | 338059 | 1522 | 1175 | n.d. | 28 | 8 | n.d. |
|  |  | **3** | 20186 | 451 | 71 | 56 | 4 | n.d. | n.d. |
|  |  | **4** | n.d. | 410 | n.d. | 33 | n.d. | n.d. | n.d. |
|  |  | **5** | n.d. | 982 | n.d. | 180 | n.d. | n.d. | n.d. |
|  |  | **sum** | 362605 | 3491 | 1266 | 269 | 32 | 20 |  |
|  | **aqua regia** | **average** | 357168 | 5952 | 1297 | 538 | 29 | 10 |  |
|  |  |  |  |  |  |  |  |  |  |
| **C-68** | **Na-acetate** | **1** | 2017 | 203 | 9 | n.d. | n.d. | 314 | 37 |
|  |  | **2** | 231757 | 644 | 405 | 23 | 16 | 23 | 13 |
|  |  | **3** | 23595 | 194 | 43 | 68 | 1.5 | 4 | 6 |
|  |  | **4** | n.d. | 131 | n.d. | n.d. | n.d. | 6 | n.d. |
|  |  | **5** | n.d. | 721 | n.d. | 666 | 7 | 3 | n.d. |
|  |  | **sum** | 257368 | 1892 | 457 | 758 | 24 | 351 | 56 |
|  | **NH4-acetate** | **1** | 6164 | 220 | 15 | n.d. | n.d. | 347 | 38 |
|  |  | **2** | 225375 | 474 | 382 | n.d. | 12 | 16 | n.d. |
|  |  | **3** | 836 | 82 | 4 | 47 | 0.2 | 11 | 4 |
|  |  | **4** | n.d. | 69 | n.d. | 23 | n.d. | 5 | n.d. |
|  |  | **5** | n.d. | 358 | n.d. | 375 | 3 | 3 | n.d. |
|  |  | **sum** | 232375 | 1204 | 400 | 445 | 15 | 382 | 41 |
|  | **aqua regia** | **average** | 252381 | 2924 | 460 | 1387 | 26 | 330 | 61 |

|  |  |  |  |  |  |  |  |  |  |
| --- | --- | --- | --- | --- | --- | --- | --- | --- | --- |
| **GBW-07120** | **Na-acetate** | **1** | 1330 | 16 | 3 | n.d. | n.d. | 1 | n.d. |
|  |  | **2** | 361128 | 1110 | 94 | 79 | 17 | 5 | n.d. |
|  |  | **3** | 28760 | 319 | 8 | 48 | 3 | n.d. | n.d. |
|  |  | **4** | n.d. | n.d. | n.d. | 97 | n.d. | n.d. | n.d. |
|  |  | **5** | n.d. | 61 | n.d. | 191 | n.d. | n.d. | n.d. |
|  |  | **sum** | 391218 | 1506 | 106 | 414 | 20 | 6 |  |
|  | **NH4-acetate** | **1** | 4362 | 31 | 3 | n.d. | n.d. | n.d. | n.d. |
|  |  | **2** | 338850 | 978 | 85 | 31 | 15 | n.d. | n.d. |
|  |  | **3** | 6691 | 223 | 2.4 | 36 | 2 | n.d. | n.d. |
|  |  | **4** | n.d. | 94 | n.d. | 80 | n.d. | n.d. | n.d. |
|  |  | **5** | n.d. | 50 | n.d. | 129 | n.d. | n.d. | n.d. |
|  |  | **sum** | 349902 | 1377 | 91 | 275 | 16 |  |  |
|  | **aqua regia** | **average** | 368675 | 3076 | 92 | 1037 | 18 |  |  |
|  |  |  |  |  |  |  |  |  |  |
| **PS-14** | **Na-acetate** | **1** | 18831 | 27 | 71 | n.d. | 1 | 3 | n.d. |
|  |  | **2** | 55697 | 990 | 304 | 86 | 68 | 5 | n.d. |
|  |  | **3** | 4145 | 242 | 21 | 4698 | 19 | 2 | 1 |
|  |  | **4** | n.d. | n.d. | n.d. | 6418 | 34 | 17 | 4 |
|  |  | **5** | 300 | 382 | 3 | 10388 | 17 | 5 | n.d. |
|  |  | **sum** | 78972 | 1641 | 398 | 21590 | 138 | 32 | 5 |
|  | **NH4-acetate** | **1** | 18164 | 9 | 69 | n.d. | 0.3 | 2 | n.d. |
|  |  | **2** | 56581 | 1060 | 311 | 33 | 64 | n.d. | n.d. |
|  |  | **3** | 3570 | 177 | 21 | 4986 | 20 | 2 | 1 |
|  |  | **4** | n.d. | 50 | 2 | 5587 | 30 | 16 | n.d. |
|  |  | **5** | 256 | 360 | 2 | 9630 | 16 | 5 | n.d. |
|  |  | **sum** | 78571 | 1657 | 404 | 20236 | 130 | 25 | 1 |
|  | **aqua regia** | **average** | 92579 | 2838 | 487 | 30468 | 169 | 32 |  |
